# Common Variants of *KCNJ10* Are Associated with Susceptibility and Anti-Epileptic Drug Resistance in Chinese Genetic Generalized Epilepsies

**DOI:** 10.1371/journal.pone.0124896

**Published:** 2015-04-13

**Authors:** Yong Guo, Kui Po Yan, Qiang Qu, Jian Qu, Zi Gui Chen, Tao Song, Xiang-Ying Luo, Zhong-Yi Sun, Chang-Long Bi, Jin-Fang Liu

**Affiliations:** 1 Department of Neurosurgery, Xiangya Hospital, Central South University, 410008, Changsha, China; 2 Department of Cardiology, the First Affiliated Hospital of Henan Collede of TCM, 450008, Zhengzhou, China; 3 Department of Pharmacology, Xiangya Hospital, Central South University, 410008, Changsha, China; 4 Institute of Clinical Pharmacology, Central South University, 410008, Changsha, China; Tor Vergata University of Rome, ITALY

## Abstract

To explore genetic mechanism of genetic generalized epilepsies (GGEs) is challenging because of their complex heritance pattern and genetic heterogeneity. *KCNJ10* gene encodes Kir4.1 channels and plays a major role in modulating resting membrane potentials in excitable cells. It may cause GGEs if mutated. The purpose of this study was to investigate the possible association between *KCNJ10* common variants and the susceptibility and drug resistance of GGEs in Chinese population. The allele-specific MALDI–TOF mass spectrometry method was used to assess 8 single nucleotide polymorphisms (SNPs) of *KCNJ10* in 284 healthy controls and 483 Chinese GGEs patients including 279 anti-epileptic drug responsive patients and 204 drug resistant patients. We found the rs6690889 TC+TT genotypes were lower frequency in the GGEs group than that in the healthy controls (6.7% *vs* 9.5%, *p* = 0.01, OR = 0.50[0.29–0.86]). The frequency of rs1053074 G allele was lower in the childhood absence epilepsy (CAE) group than that in the healthy controls (28.4% *vs* 36.2%, *p* = 0.01, OR = 0.70[0.53–0.93]). The frequency of rs12729701 G allele and AG+GG genotypes was lower in the CAE group than that in the healthy controls (21.2% *vs* 28.4%, *p* = 0.01, OR = 0.74[0.59–0.94] and 36.3% *vs* 48.1%, *p* = 0.01, OR = 0.83[0.72–0.96], respectively). The frequency of rs12402969 C allele and the CC+CT genotypes were higher in the GGEs drug responsive patients than that in the drug resistant patients (9.3% *vs* 5.6%, OR = 1.73[1.06–2.85], *p* = 0.026 and 36.3% *vs* 48.1%, *p* = 0.01, OR = 0.83[0.72–0.96], respectively). This study identifies potential SNPs of *KCNJ10* gene that may contribute to seizure susceptibility and anti-epileptic drug resistance.

## Introduction

Epilepsy is one of the most common neurological disorders characterized by recurring unprovoked epileptic seizures and caused by synchronized electrical discharges of central neurons[1].Seizure disorders always be divided into idiopathic or symptomatic epilepsies. Genetic generalized epilepsies (GGEs, also called the idiopathic generalized epilepsies) are characterized by non-focal mechanism of onset and no external cause which affects about 0.3% of the general population and accounts for 30% of all epilepsies[2]. GGEs has some other subtypes including childhood absence epilepsy (CAE), juvenile absence epilepsy (JAE), juvenile myoclonic epilepsy (JME) and epilepsy with generalized tonic-clonic seizures alone (EGTCS) [1]. In recent years, there have been important advances in understanding the genetic basis of GGEs. Many of them are channelopathies[3], but only a small fraction of cases have been determined.

*KCNJ10* gene, which is located at chromosome 1q22–23 coding for inward rectifier potassium ion channel protein (Kir4.1) is highly expressed in various tissues, including inner ear, eye, and kidney, especially in the brain[4, 5]. The role of KCNJ10 is to recycle potassium, which is necessary for the function of the primary active Na+/K+-ATPase[6]. Moreover, Kir4.1 plays an important role in maintaining resting membrane potential by transporting potassium from the extracellular space into glial cells in the CNS[7, 8]. As a gene encoding potassium channel, *KCNJ10* may not only be related to the susceptibility of GGEs, but also be related to the efficacy of anti-epileptic drugs(AEDs). It is the electrophysiological basis state of nerve cells. And different electrophysiological basis state of nerve cells may respond differently to AEDs.

Recent studies showed that the expression of astrocytic Kir4.1 channels is elevated in a pilocarpine-induced rat model of temporal lobe epilepsy. Reduced activity of Kir4.1 channels in astrocytes of mice is associated with deficits in potassium and glutamate buffering[9, 10]. Genetic linkage studies have indicated a linkage between missense variants in Kir4.1 and seizure susceptibility [11, 12]. Some studies found that *KCNJ10* gene rs1130183 (Arg271Cys) was associated with seizure resistance in groups of patients with both focal and generalized epilepsy[11, 13].It is also suggested that a missense variant (Thr262Ser) in *KCNJ10* was likely to be candidate gene for seizure[12]. The *KCNJ10* gene rs2486253 polymorphism affects risk for development of common types of childhood epilepsy. The T allele of this polymorphism was found to be a seizure-susceptibility allele for tonic-clonic epilepsy[14]. All there evidences are consistent with the role of the *KCNJ10* variants in the pathogenesis of some rare epileptic syndromes[15].

Therefore, we hypothesized that *KCNJ10* common variants may be related with the susceptibility and drug resistance of GGEs in Chinese population. Thus, we analyzed eight tagSNPs of *KCNJ10* in 483 Chinese GGEs patients and 284 healthy controls to further investigate whether the common variants of *KCNJ10* are involved in the etiology of GGEs and AEDs resistance.

## Methods and Materials

### Subjects

483 GGEs patients treated with AEDs and 284 healthy controls from Xiangya hospital, the Second Xiangya Hospital of Central South University and Hunan Provincial People’s Hospital were recruited in this study. The patients were diagnosed and classified according to guidelines of the International League Against Epilepsy and standardized protocols[16, 17]. Inclusion and exclusion criteria were shown in **S1 Table**. A standardized questionnaire was administered to collect clinical details such as seizure types and frequency, past medical history, AED history, concomitant drug history and relevant family history etc. All adult patients or children’s parents gave their written consent to participate in the study. All the patients were provided written informed consent in compliance with the code of ethics of the World Medical Association (Declaration of Helsinki) before this study was initiated. The study protocol was approved by the Ethics Committee of Xiangya School of Medicine and Ethics Committee of Central South University. Clinical study admission (the registration number: ChiCTR-RO-12002853) was approved by Chinese Clinical Trail Register. The patients were considered to be drug-responsive if they had not experienced any type of seizures for a minimum of 1 year after receiving AEDs. Drug resistance was defined as having at least four seizures during the previous year while trying at least three antiepileptic medications at maximal tolerated doses[18, 19].

### Genotyping

Blood samples (3 ml) for genotyping were obtained with EDTA through the venipuncture and frozen at -80°C for 24 hours. DNA was isolated using phenol-chloroform extraction method. Linkage disequilibrium (LD) data for SNPs with minor allele frequency (MAF) ≧0.1 in Han Chinese, Beijing, China from the International HapMap Project (http://www.hapmap.org) were used with tagger to identify SNPs tagging clusters with LD of r^2^>0.8. Seven tagged-single nucleotide polymorphisms (tagSNPs) were identified across the gene regions of *KCNJ10* (31.93 kilobase pairs [kbp], HapMap Data Rel 24/phaseⅡNov08, on NCBI B36 asembly dbSNP b126) by Haploview (http://www.broad.mit.edu/mpg/haploview). A missense mutation rs1130183 was also added to perform in our study, which is reported to relate with epilepsy[13]. MassArray (Sequenom, SanDiego, CA) was used for genotyping all tagSNPs using allele-specific MALDI–TOF mass spectrometry. Primers and multiplex reactions were designed using the RealSNP.com Website. The genotyping call rate was larger than 96% for all SNPs.

### Statistical analysis

The SPSS software package (Version 13.0 for Windows; SPSS, Chicago, IL, USA) was used for statistical analysis. Hardy-Weinberg equilibrium was analyzed with χ^2^ test or Fisher’s Exact test as applicable in the studied samples. Age was compared between responsive, non-responsive patients or healthy controls with the Student’s t-test. Sex, allele and genotype frequencies between cases and controls by χ^2^ test. The relationship between various genotypes and anti-epileptic drug efficacy was examined by binary logistic regression, after adjustment for age, sex and seizure types. Statistical significance was accepted when *P* < 0.05.

## Results

**Fig 1:**
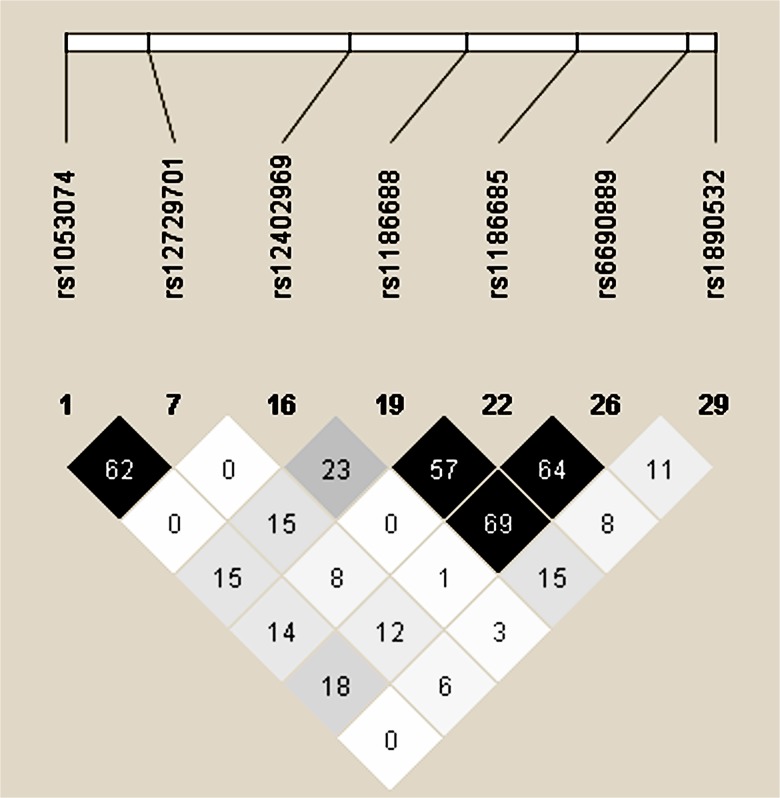
Linkage disequilibrium of 7 tagSNPs in *KCNJ10*. Linkage disequilibriums between pairs of polymorphisms are shown with diamonds (r^2^), with darker shading indicating greater r^2^.

The study population consisted of 284 healthy controls (184 males, 100 female, mean age: 18.6 ± 12.2 years) and 483 GGEs Chinese patients treated with AEDs (297 males, 186 female, mean age: 18.3 ± 12.1 years), including 279 drug responsive patients and 204 drug resistant patients. In all 483 Chinese GGEs patients, the subtypes of GGEs contained CAE (38.3%), JAE (19.6%), JME (25.6%) and EGTCS (16.5%) (**Table 1**). There were no difference on sex and age between controls and GGEs. Moreover, there was also no difference on sex, age and age at onset between drug responsive patients and drug resistant patients. Anti-epileptic drugs (AEDs) in drug-responsive and drug-resistant GGEs patients were shown in **Table 2**. Seven tagSNPs and one missense mutation were chosen based on a comprehensive study of all tagSNPs across the entire *KCNJ10* regions using HapMap data and the Haploview software (**Table 3 and Fig 1**).

**Table 1:** Demographic and clinical characteristics of the drug-responsive and drug-resistant GGE patients and the healthy controls.

Parameter	Drug- responsive (279)	Drug- resistant (204)	Total (483)	Healthy controls (284)
Male/ Female	174(62.4%)/105(37.6%)	123(60.3%)/81(39.7%)	297(61.5%)/186(38.5%)	184(64.8%)/100(35.2%)
Age (years)	19.3±12.2	17.7±11.9	18.3±12.1	18.6 ± 12.2
age at onset(years)	10.6±8.9	9.6±10.2	10.3±10.1	-
Seizure type (%)				
CAE	120(40.4%)	65(31.9%)	185(38.3%)	
JAE	55(19.7%)	39(19.1%)	94(19.4%)	
JME	67(22.6%)	57(27.9%)	124(25.7%)	
EGTCS	37(13.3%)	43(21.1%)	80(16.6%)	

GGE, genetic generalized epilepsies; CAE, childhood absence epilepsy; JAE, juvenile absence epilepsy; JME, juvenile myoclonic epilepsy; EGTCS, epilepsy with generalized tonic-clonic seizures.

**Table 2:** Anti-epileptic drugs (AEDs) in drug-responsive and drug-resistant GGEs patients.

AEDs	Drug- responsive(279)	Drug- resistant(204)	Total(483)
Carbamazepine	174(62.1%)	137(67.2%)	310(62.3%)
Valproic acid	182(65.0%)	168(82.4%)	350(72.5%)
Phenytoinum	23(8.2%)	18(8.8%)	41(8.5%)
Phenobarbital	13(4.6%)	8(3.9%)	20(4.1%)
lamotrigine	39(13.9%)	28(13.7%)	67(13.9%)
Levetiracetam	29(10.6%)	19(9.3%)	48(9.9%)
Topiramate	44(15.7%)	23(11.3%)	67(13.9%)
Oxcarbazepine	57(20.4%)	25(12.3%)	82(17.0%)

**Table 3:** Positions and location of *KCNJ10* all tagSNPs.

Rs Number	SNP	Position	Location
rs1053074	G/T	158275745	Intron
rs1130183	C/T	158278136	Exon
rs12729701	A/G	158279030	Intron
rs12402969	C/T	158286861	Intron
rs1186688	T/C	158291507	Intron
rs1186685	T/C	158295836	Intron
rs6690889	T/C	158300126	Intron
rs1890532	C/G	158301199	Intron

SNP locations are based on a comprehensive study of all tagSNPs across the entire KCNJ10 gene regions that was conducted with HapMap data and the Haploview software.SNP, single-nucleotide polymorphism.

We tested the frequencies of these eight SNPs in 483 GGEs Chinese patients and 284 healthy controls (**Table** 4). The investigated SNPs were all in Hardy-Weinberg equilibrium in case-controls except missense mutation rs1130183. We found one homozygous mutation of rs1130183 in GGEs group and none mutation in controls. There were no significant difference between two groups about rs1130183 allelic and genotype distribution. We found rs6690889 TC+TT genotypes were less frequent in GGEs group than that in healthy controls (6.7% *vs* 9.5%, *p* = 0.01, OR = 0.50[0.29–0.86]). Moreover, we also analyzed the association in four subtypes of GGEs. The frequency of rs1053074 G allele was lower in CAE group than that in healthy controls (28.4% *vs* 36.2%, *p* = 0.01, OR = 0.70[0.53–0.93]). The frequencies of rs12729701 G allele and AG+GG genotypes was lower in CAE group than that in healthy controls (21.2% *vs* 28.4%, *p* = 0.01, OR = 0.74[0.59–0.94] and 36.3% *vs* 48.1%, *p* = 0.01, OR = 0.83[0.72–0.96], respectively) (**Table 5**).

**Table 4:** Allelic and genotypic frequencies of 8 SNPs of KCNJ10 in the GGEs patients (n = 483) and the healthy controls (n = 284).

		Genotypes							Alleles		
		GGEs patients			Healthy controls				GGEs A:B		
SNPs	Alleles	AA	AB	BB	AA	AB	BB	p-value	Control A:B	OR(95% CI)	p-value
rs1053074	G/T	58	200	224	44	116	122	0.35	316648; 204/360	0.86(0.69–1.07)	0.18
rs1130183	C/T	482	0	1	284	0	0	0.44	964/2;568/0	-	0.28
rs12729701	A/G	282	160	35	147	111	25	0.16	724/230 ;405/161	1.25(0.99–1.58)	0.06
rs12402969	C/T	5	59	418	2	40	241	0.68	69/895;44/522	0.92(0.62–1.36)	0.66
rs1186688	T/C	42	208	231	34	115	134	0.33	292/670;183/383	0.91(0.73–1.14)	0.42
rs1186685	T/C	21	175	285	22	96	166	0.14	217/745;140/428	0.89(0.70–1.14)	0.35
rs6690889	T/C	264	193	23	145	111	27	**0.04**	721/239;401/165	1.24(0.98–1.57)	0.07
rs1890532	C/G	299	158	24	182	85	15	0.74	756/2065;449/115	0.94(0.73–1.21)	0.64

OR, odds ratio; CI, confidence interval; GGEs, genetic generalized epilepsies.

**Table 5:** Significant difference on allelic and genotypic frequencies of *KCNJ10* in the GGEs patients (n = 483)/the CAE patients (n = 185) and the healthy controls (n = 284).

SNP	Patients	Controls	*p*-value	OR (95% CI)
rs6690889	T 721 (0.751)	T 401 (0.708)		
(T/C)	C 239 (0.249)	C 165(0.292)	0.07	1.24[0.98–1.57]
	TT 457(0.933)	TT 256(0.905)		
	TC+TT 23(0.067)	TC+TT 27(0.095)	**0.01**	**0.50[0.29–0.86]**
rs1053074	T 265(0.716)	T 360(0.638)		
(G/T)	G 105(0.284)	G 204(0.362)	**0.01**	**0.70[0.53–0.93]**
	TT 97(0.524)	TT 122(0.433)		
	GT+GG 88(0.476)	GT+GG 160(0.567)	0.052	0.86[0.74–1.00]
rs12729701	A 287(0.788)	A 405(0.716)		
(A/G)	G 77(0.212)	G 161(0.284)	**0.01**	**0.74[0.59–0.94]**
	AA 116(0.637)	AA 147(0.519)		
	AG+GG 66(0.363)	AG+GG 136(0.481)	**0.01**	**0.83[0.72–0.96]**

GGE, genetic generalized epilepsies; CAE, childhood absence epilepsy; JAE, juvenile absence epilepsy; JME, juvenile myoclonic epilepsy; EGTCS, epilepsy with generalized tonic-clonic seizures.

**Fig 2:**
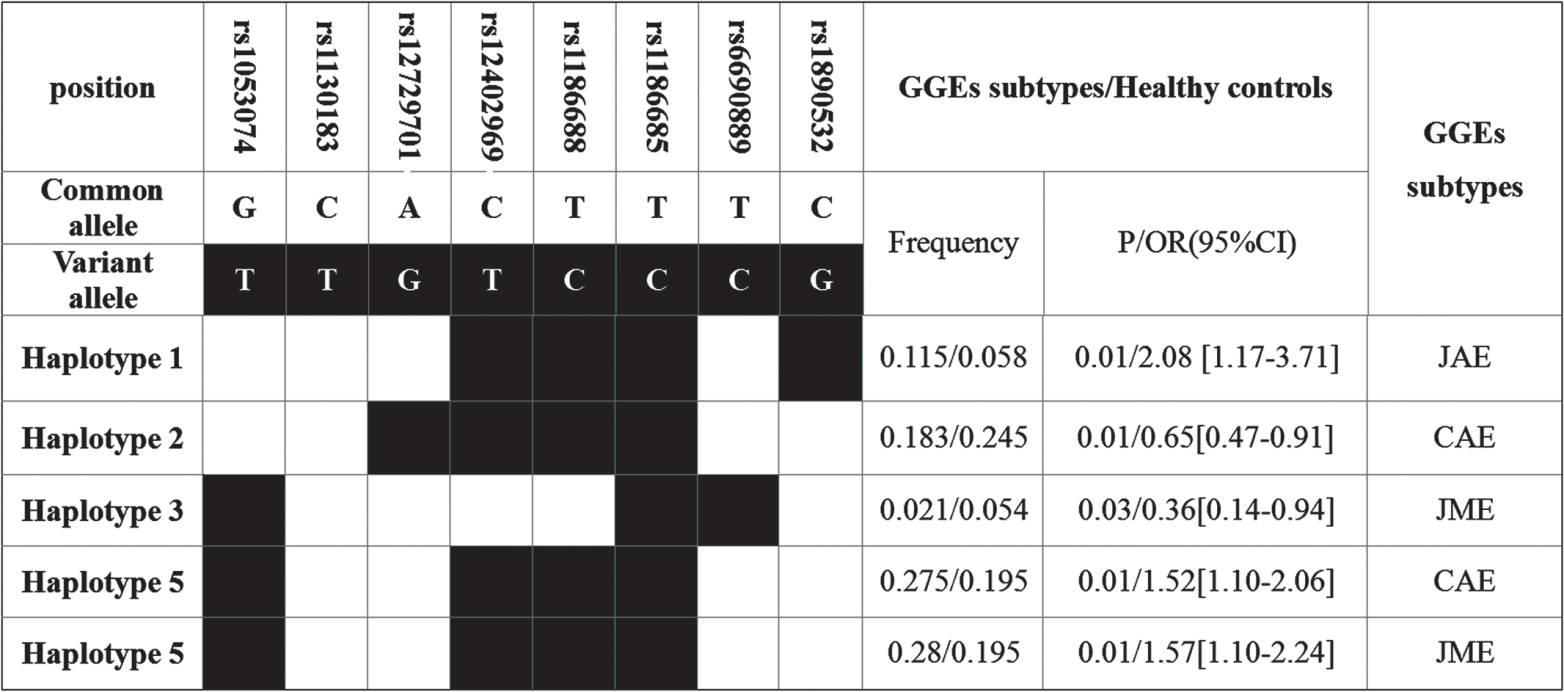
Frequencies of the haplotypes (>3%) containing all of the tagSNPs of *KCNJ10* in the drug-resistant (n = 204) and drug-responsive GGE patients (n = 279).

**Fig 3:**
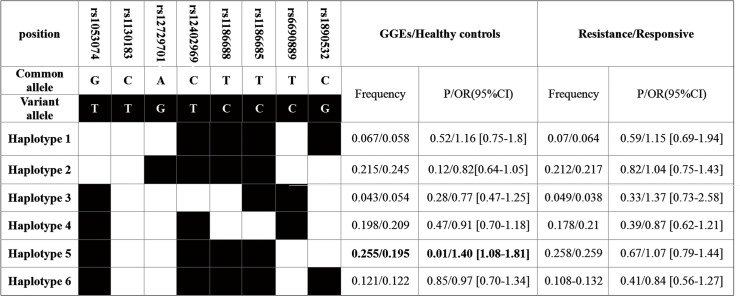
Significant different frequencies of *KCNJ10* haplotypes between the GGEs subtypes and the healthy controls.

We analyzed the *KCNJ10* haplotypes frequencies in GGEs or its subtypes and in healthy controls. We found the frequency of a haplotype (haplotype 5) was significant higher in GGEs than that in in healthy controls (25.5% *vs* 19.5%, OR = 1.40 [1.08–1.81], *p* = 0.01) (**Fig 2**). Two haplotypes were associated with CAE and JME, respectively and one haplotype was associated with JAE (**Fig 3**).

By analyzing the association between KCNJ10 polymorphisms and anti-epileptic drug efficacy of GGEs we found the frequency of rs12402969 C allele and CC+CT genotypes were higher in GGEs drug responsive patients than that in drug resistant patients (9.3% vs 5.6%, OR = 1.73[1.06–2.85], p = 0.026 and 36.3% vs 48.1%, p = 0.01, OR = 0.83[0.72–0.96], respectively) (Table 6). In haplotypes calculation, we found no haplotypes was associated with drug resistance (Fig. 2). For the limited sample size and small number of SNPs which were analyzed, no further correction for multiple testing has been performed. Therefore less false negative would not be dropped. It is a limitation of our study.

**Table 6:** Allelic and genotypic frequencies of 8 SNPs of *KCNJ10* in the drug-responsive (n = 279) and drug-resistant GGEs patients (n = 204).

		Genotypes							Alleles		
		Drug-responsive			Drug-resistant				GGEs A/B		
SNPs	Alleles	**AA**	**AB**	**BB**	**AA**	**AB**	**BB**	**p-value**	**Control A/B**	**OR(95% CI)**	**p-value**
rs1053074	G/T	27	85	91	31	115	133	0.71	139/267; 177/381	1.12[0.85–1.47]	0.41
rs1130183	C/T	202	0	1	278	0	0	0.24	404/2;556/0	-	0.1
rs12729701	A/G	115	73	13	167	87	22	0.5	303/99 ;421/131	0.95[0.71–1.29]	0.75
rs12402969	C/T	3	32	169	2	27	249	0.096	38/370;31/525	**1.73[1.06–2.85]**	**0.026**
rs1186688	T/C	16	92	95	26	116	136	0.69	124/282;168/388	1.02[0.77–1.34]	0.91
rs1186685	T/C	4	76	124	17	99	161	0.09	84/324;133/421	0.82[0.60–1.12]	0.21
rs6690889	T/C	113	82	7	151	111	16	0.51	308/96;413/143	1.11[0.82–1.50]	0.49

OR, odds ratio; CI, confidence interval; GGEs, genetic generalized epilepsies; OR, odds ratio estimated with binary logistic regression analysis and adjusted for age, age of epilepsy onset and gender.

## Discussion

Although more and more research focused on the pathogenesis of epilepsy, especially on the GGEs, little is known due to its genetic complexity and heterogeneity. To date, the technology of Genome-wide linkage scans and Genome-wide copy number variation shed some lights to finding the susceptibility loci regions, but the real susceptibility genes still hide in darkness [16, 20, 21]. In contrast to the positional gene mapping strategies, lots of small-scale linkage and candidate gene association studies failed to identify replicable susceptibility genes for common GGE syndromes[22, 23]. Studies about the relationship between *KCNJ10* polymorphisms and epilepsy were inconsistent[11, 13, 24]. Moreover, there were no research about the tagSNP of *KCNJ10* and subtypes of GGEs, *KCNJ10* polymorphisms and AEDs resistance.

Hence we performed the study that involved 483 GGEs Chinese patients and 284 healthy controls, and detected the eight SNPs of *KCNJ10*, then analyzed the association between the susceptibility of genetic generalized epilepsies. We found that rs6690889 was associated with the susceptibility of GGEs; the rs1053074 and the rs12729701 were associated with CAE.

*KCNJ10* encodes the potassium channel Kir4.1 whose function is to recycle potassium, which is necessary for the primary active Na+/K+-ATPase[15]. The mutations of *KCNJ10* cause specific disorders, consisting of epilepsy, ataxia, sensorineural deafness, and tubulopathy[8, 15, 25, 26]. Among previous studies focused on the association between common variants of *KCNJ10* and seizure susceptibility, some studies were positive results[11, 14, 27]. However, some studies were negative results[13, 28]. The positive results suggest that SNP rs1130183 (C > T) alters amino acid 271 from an arginine to a cysteine (R271C) and it is related to general seizure susceptibility in humans[11]. However, *KCNJ10* rs1130183 dose not contribute to risk of seizure susceptibility in Turkish or Indian patients with idiopathic generalized epilepsies[13, 14]. In our study we also replicated the association about rs1130183 but found no significant. We just found one homozygous mutation of rs1130183 in 483 patients and no mutation in 284 healthy controls. It suggests that rs1130183 (C > T) is a rare mutation and is not related to the susceptibility of epilepsy in our study. Further more, we found rs6690889 was associated with the susceptibility of GGEs; rs1053074 and rs12729701 were associated with CAE. Although the three positive SNPs are intron polymorphisms, it has the possibility that these positive SNPs may have linkage disequilibrium with other functional SNPs.

Therefore we used the HapMap data and the Haploview software to find the possible linkage disequilibrium with potential functional variants, the results showed that rs6690889 has strong linkage disequilibrium with other 7 SNPs which all are all intron polymorphisms. There were no other functional variants that have linkage disequilibrium with rs1053074 and rs12729701. When we used the hapmap data to predict, there were no functional SNPs found to be linkage disequilibrium with these positive intron polymorphisms (**S1 Fig**). It is possible that they have linkage disequilibrium with functional variants in patients’ data. Rs1053074 is localized to the 3′untranslated region of *KCNJ10*, we predicted its effect on altering miRNA target sites using MirSNP database[29]. The results showed that this SNP could influence the hsa-miR-4422, hsa-miR-548s and hsa-miR-551b-5p binding with *KCNJ10* thus modulating the expression of KCNJ10 (**S2 Fig**). The functional impact of this polymorphism on *KCNJ10* needs further validation.

Nowadays, lots of anti-epileptic drugs were used to control seizure, but there are still 30% of patients with epilepsy continue to have seizures after using AEDs[30]. It is becoming increasingly clear that genetic polymorphisms play an integral role in variability of AEDs pharmacodynamics[31]. Although presently available AEDs appear to be directed against a relatively small number of targets (mainly ion channels or other components of the synaptic machinery), it is complicated to find the real drug resistance by the fact that many AEDs seem to work via multiple mechanisms and some of which are still unresolved. The different pathogens of epilepsy may influence the efficacy of anti-epileptic drugs. Different electrophysiology baseline of epileptic patients may have different efficacy of AEDs. In our study, we used the genetic generalized epileptic patients as study objects to avoid the interference of different epileptic causes. We performed our studies on the association between *KCNJ10* common variants and AEDs’ efficacy in 279 drug responsive Chinese GGEs patients and 204 drug resistant patients. We found rs12402969 was related to drug resistance. Although it is located in intron region of *KCNJ10* gene, there is the possibility of linkage disequilibrium for functional SNPs. We used the HapMap data and the Haploview software to find the possible linkage disequilibrium with potential functional variants. Although failed, it is possible that the SNP may be linkage disequilibrium with functional variants in patients’ data.

Epilepsy has many complex phenotype-genotype subtypes and was thought to be the outcome of ploygenic-environment interactions. Up to now, lots of epileptic patients cannot find the real causes. Moreover, there were no precise evaluation criteria of AEDs efficacy. Thirdly, the relatively small study samples and investigated SNPs of *KCNJ10* and no further correction for multiple testing has been performed. All these aspects limited the results of our research. In our studies, we performed a gene-wide tagging study of the association between *KCNJ10* tagSNPs and the susceptibility/AEDs efficacy of genetic generalized epilepsy in Chinese population. We found that some *KCNJ10* tagSNPs were associated with the susceptibility and efficacy of genetic generalized epilepsy. Further studies on how these SNPs impact the pathogenesis of GGEs and AEDs resistance should be warranted.

## Supporting Information

S1 Table The Inclusion criteria and exclusion criteria of genetic generalized epilepsies in this study. (DOCX)

S1 Fig Linkage disequilibrium of 7 tagSNPs and other SNPs in *KCNJ10*. Linkage disequilibriums between pairs of polymorphisms are shown with diamonds (r^2^), with darker shading indicating greater r^2^.
(TIF)

S2 Fig *KCNJ10* rs1053074 function prediction in MirSNP database. (TIF)
