# Supplementary material for: Common Variants of KCNJ10 Are Associated with Susceptibility and Anti-Epileptic Drug Resistance in Chinese Genetic Generalized Epilepsies
Source: PLoS One. 2015 Apr 13;10(4):e0124896. doi: 10.1371/journal.pone.0124896 (PMC4395153; doi:10.1371/journal.pone.0124896)
Supplement: S1 Table — (DOCX) [file pone.0124896.s001.docx]

Inclusion criteria: normal intelligence, psychomotoric development, normal neurologic examination status and EEG: generalised spike-wave discharges (2.5–5 Hz), normal background activity.

Exclusion criteria: structural, metabolic or degenerative brain disorders, exclusively stimulus-induced seizures, mental retardation, severe adverse drug reactions, unreliable record of seizure frequency, poor compliance with AEDs, history of alcohol or drug abuse, presence of progressive or degenerative neurological or systemic disorders, and hepatic or renal failure.
