# Supplementary figures and images for: Common Variants of KCNJ10 Are Associated with Susceptibility and Anti-Epileptic Drug Resistance in Chinese Genetic Generalized Epilepsies
Source: PLoS One. 2015 Apr 13;10(4):e0124896. doi: 10.1371/journal.pone.0124896 (PMC4395153; doi:10.1371/journal.pone.0124896)

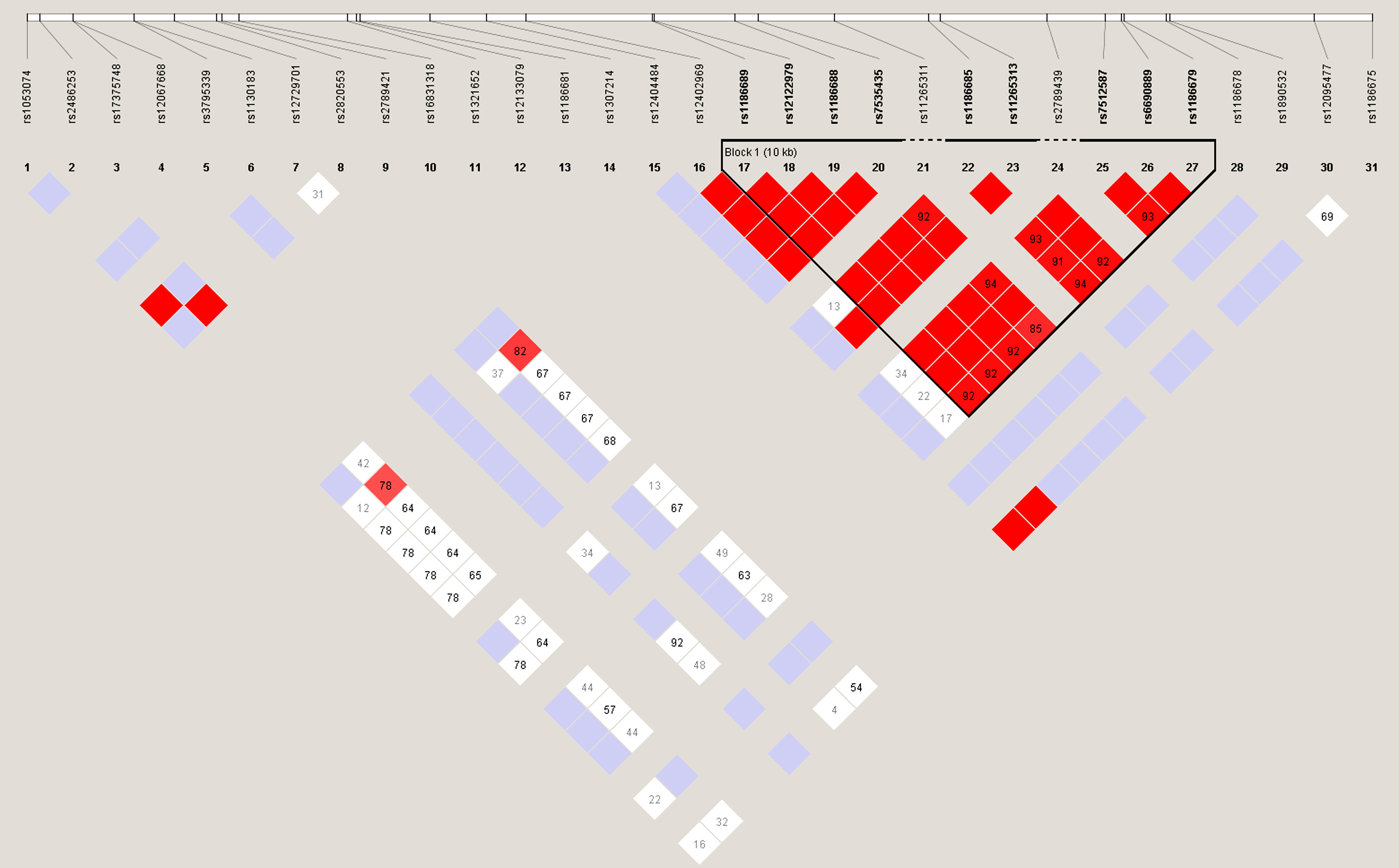

Supplement: S1 Fig — Linkage disequilibriums between pairs of polymorphisms are shown with diamonds (r2), with darker shading indicating greater r2. (TIF) [file pone.0124896.s002.tif]

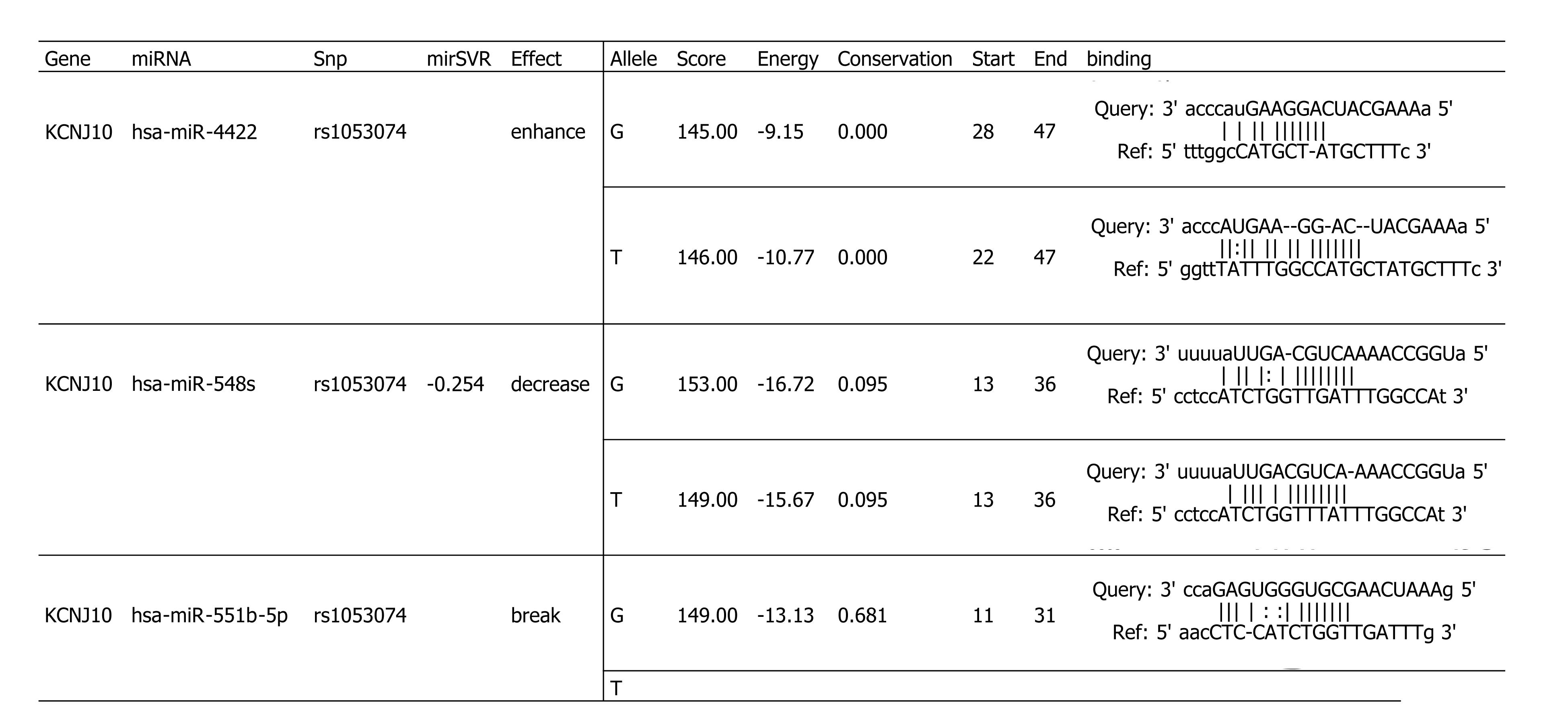

Supplement: S2 Fig — (TIF) [file pone.0124896.s003.tif]
